# Association of Vitamin D Deficiency With Abnormal Electrocardiographic Activity Among Healthy People

**DOI:** 10.1002/fsn3.70340

**Published:** 2025-06-06

**Authors:** Lin Yang, Jinsen Wang, Xiaoyu Pan, Xueqing Zhang, Jiangli Ban, Lin Yue, Lin Ren, Xiaolan Jiang, Shuchun Chen

**Affiliations:** ^1^ Department of Internal Medicine Hebei North University Zhangjiakou Hebei People's Republic of China; ^2^ Life Sciences Research Center Hebei North University Zhangjiakou Hebei People's Republic of China; ^3^ Department of Endocrinology Hebei General Hospital Shijiazhuang Hebei People's Republic of China; ^4^ Department of Internal Medicine Hebei Medical University Shijiazhuang Hebei People's Republic of China; ^5^ Department of Endocrinology The Third Hospital of Shijiazhuang Shijiazhuang Hebei People's Republic of China

**Keywords:** heart rate variability, ventricular repolarization, vitamin D deficiency

## Abstract

In a healthy population, abnormal electrocardiographic activity is linked to an increased risk of cardiovascular events. The connection between vitamin D (VitD) deficiency and aberrant electrocardiographic activity was investigated. We included all papers that looked at the association between VitD deficiency and electrocardiographic activity. Basic information about the research population was gathered, and the quality of the studies that were included was evaluated. Using forest plots, we determined the impact of VitD deficiency on cardiac repolarization and autonomic function. A total of 6 studies with a total of 532 participants were included in the study. VitD deficiency was found in 197 people, whereas VitD insufficiency was found in 63 people, and VitD sufficiency was found in 272. When compared to those who had sufficient VitD, VitD deficiency considerably reduced PNN50, SDANN, and RMSSD (*p* < 0.05), slightly lowered SDNN (*p* = 0.05), and lengthened TP‐e (*p* < 0.05). Heart rate, QRS, QTc, JTc, HF, LF, and RMSDD, on the other hand, did not differ substantially between the two groups (*p* > 0.05). Insufficient VitD does not appear to be linked to aberrant electrocardiographic activity. VitD deficiency affects heart rate variability (HRV) and ventricular repolarization indexes compared to those with sufficient VitD. VitD insufficiency does not affect the ventricular repolarization index.

## Graphical abstract

This study examined the relationship between vitamin D (VitD) deficiency and abnormal electrocardiograms. Compared with the VitD‐sufficient group, the VitD‐deficient group exhibited significantly lower values for PNN50, SDANN, and RMSSD, as well as slightly reduced SDNN and significantly prolonged TP‐e intervals. No significant differences were observed in other indicators between the two groups. These findings suggest that vitamin D deficiency may not be directly associated with abnormal electrocardiographic activity.

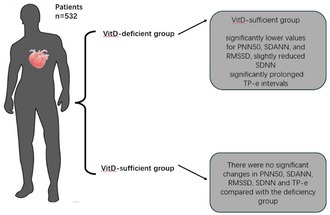

**Abbreviations**
HF: High‐frequency component
HRV: Heart rate variability
JT‐c: Corrected QRS end (J point) and T wave end distance
LF: Low‐frequency component
NOS: Newcastle‐Ottawa Scale
PNN50: The proportion of differences in successive NN intervals greater than 50 ms
QTc: Corrected QT
QTd: QT dispersion
RMSSD: Square root of the mean of the sum of the squares of differences between adjacent RR intervals
SDANN: Standard deviation of the 5‐min mean RR intervals tabulated over an entire day
SDNN: Standard deviation of all NN intervals for a selected time period
TP‐e: T wave end time with the peak point of the T wave
VDr: Vitamin D receptor
VitD: Vitamin D

## 1 Introduction

Vitamin D (VitD), a steroid hormone and a product of steroids, is derived from food and absorbed mostly by fat absorption in the jejunum and ileum. VitD deficiency or insufficiency is a frequent problem all over the world. According to observational research, approximately 40% of Europeans have insufficient VitD levels, whereas more than 20% of Indians, Afghans, and Pakistanis have severe VitD deficiency (Lips et al. 2019). Low levels of VitD have been linked to an increased risk of cancer, cardiovascular disease, and neurological illnesses (Pines 2014). Reduced VitD levels are closely caused by obesity, dyslipidemia, and diabetes (Han et al. 2021).

Recent research has found that VitD deficiency affects cardiac electrical activity and cardiac autonomic function in the context of a variety of underlying illnesses, increasing the risk of lethal arrhythmias and sudden cardiac death (Luo et al. 2022; Yaman et al. 2020). However, sudden cardiac death is becoming more common in people who have no obvious organic disease, which could be linked to cardiac autonomic dysfunction. The noninvasive and reliable indicators of the electrical activity of the heart are heart rate variability (HRV) (cardiac autonomic index) and ventricular repolarization. HRV is an indicator of autonomic nervous system activity and quantitative assessment of cardiac sympathetic and vagal tone and its balance, and may be a valuable predictor of sudden cardiac death and arrhythmic events. The main indicators of HRV measurement include SDNN, SDANN, RMSSD, RMSDD, LF, HF, PNN50 (Tiwari et al. 2021). Abnormalities in ventricular repolarization found on electrocardiogram can predict malignant ventricular arrhythmias. QRS, QTc, JTc, TP‐e can be used to indicate the presence of ventricular repolarization abnormalities (Yilmaz Coşkun et al. 2018). The connection between VitD deficiency and electrical activity indicators such as HRV and ventricular repolarization indexes could help researchers identify a link between the two that could aid the earlier detection and intervention in the healthy population, lowering the risk of sudden cardiac death and malignant arrhythmias. However, there are few studies in healthy individuals that link VitD deficiency to aberrant cardiac electrical activity, and the sample sizes are tiny, making the findings questionable.

In this meta‐analysis, we evaluated the association between VitD deficiency and aberrant electrocardiographic activity in a healthy population by considering heart rate, HRV (measured by SDNN, SDANN, RMSSD, RMSDD, LF, HF, PNN50) and ventricular repolarization (measured by QRS, QTc, JTc, TP‐e). This project is well underway to better understand the link between VitD and abnormal cardiac activity and to be able to identify and intervene earlier in this population to reduce the risk of sudden cardiac death and malignant arrhythmias.

## 2 Materials and Methods

### 2.1 Search Strategy

We conducted this investigation in compliance with the Strengthening the Reporting of Observational Studies in Epidemiology (STROBE) statement because observational studies have inherent biases and variances in study design. Until May 2022, a comprehensive literature search was carried out utilizing PubMed, EMBASE, Web of Science, and Cochrane Libraries. The terms “vitamin D,” “25(OH)D,” “arrhythmia,” “electrocardiogram,” and “heart rate” were employed. Conferences, expert consensus, and unpublished studies were also manually added. Two scholars worked independently on the literature search.

### 2.2 Inclusion and Exclusion Criteria

All observational studies on the relationship between VitD and cardiac electrical activity were included. (1) healthy population studies; (2) observational studies; (3) reporting VitD levels; and (4) reporting cardiac electrical activity markers were the study's inclusion criteria.

Exclusion criteria were as follows: (1) animal studies; (2) non‐healthy populations; (3) HRV and ventricular repolarization indices were not reported; (4) quality assessment results < 6 stars; and (5) study protocols, narrative reviews, reviews, dissertations, and conference abstracts.

### 2.3 Data Extraction and Quality Evaluation

The research was conducted using publicly available data obtained legally and therefore did not require ethical review. Two investigators independently assessed the collected literature for inclusion and exclusion criteria. The title and abstract are read first for preliminary screening; second, the full text is determined for final inclusion in the document and data extraction is performed; third, the last investigator cross‐checks, and if discrepancies are discussed with a third investigator, a third investigation is made at the discretion of the member. The Newcastle‐Ottawa Scale (NOS) was used to assess the quality of all included studies, with a NOS score of 6 stars indicating high‐quality studies and anything below indicating low‐quality studies.

### 2.4 Statistical Analysis

All data were analyzed using the reviewmanager 5.3 software. The continuity variable is represented by MD, and the interval estimation is the 95% confidence interval (CI). The heterogeneity between the studies was expressed by *I* ^2^ value. 0 ≤ *I* ^2^ < 25% suggested that there was no heterogeneity among the studies; 25% < *I* ^2^ < 50% suggested low heterogeneity; 50% < *I* ^2^ ≤ 75% suggested moderate heterogeneity; *I* ^2^ > 75% suggested high heterogeneity. The fixed effect model was used when *I* ^2^ was less than 50%. When *I* ^2^ > 50%, the cause of heterogeneity was analyzed first, and if there was no clinical heterogeneity, the random effect model was used. According to the Cochrane manual, less than 10 articles of the study were not suitable for the detection of publication bias, so no publication bias test was carried out in this study.

## 3 Results

### 3.1 Literature Search Results

**FIGURE 1:**
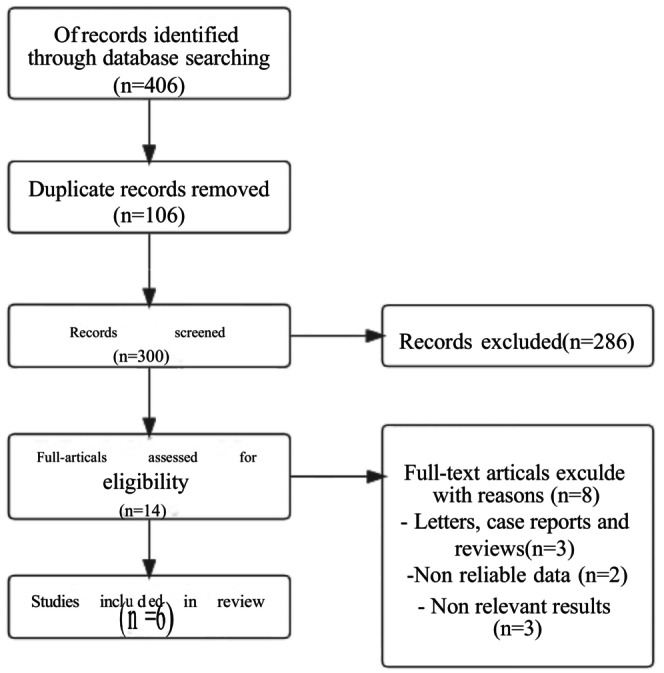
Flowchart of study selection.

The preliminary search yielded 406 articles, according to the search strategy. Six papers were chosen for further study after deleting duplicates, reading titles and abstracts, and reading the whole text (Figure 1). There were 532 people in all, including 197 cases of VitD deficiency, 63 cases of insufficient VitD, and 272 cases of sufficient VitD. Table 1 shows the main characteristics of the studies that were considered. VitD deficiency was classified as < 20 ng/mL of serum VitD, 20–30 ng/mL as VitD insufficiency, and ≥ 30 ng/mL as VitD sufficiency.

**TABLE 1:** Study characteristics.

Author	Country	Total population	Group	Population (man%)	Age	Indicators
Bagrul and Atik (2019)	Turkey	150	Deficient vitamin D	50 (52%)	14.8 ± 3.8	HR/QRS/QTc/JTc
			Insufficient vitamin D	50 (50%)	14.1 ± 3.6	
			Sufficient vitamin D	50 (50%)	14.2 ± 3.8	/TP‐e
Bekdas et al. (2021)	Turkey	67	Deficient vitamin D	10 (70%)	12.2 ± 4.1	HR/QRS/QTc/JTc
			Insufficient vitamin D	13 (38%)	10.2 ± 3.7	
			Sufficient vitamin D	44 (50%)	11 ± 2.9	/TP‐e
Dogdus et al. (2019)	Turkey	102	Deficient vitamin D	52 (46.2%)	34.6 ± 7.4	HR/SDNN/SDANN/RMSSD/PNN50/HF
			Sufficient vitamin D	50 (46%)	37.5 ± 7.6	/LF
Canpolat et al. (2015)	Turkey	74	Deficient vitamin D	24 (50%)	37.2 ± 6.73	HR/SDNN/SDANN/RMSSD/PNN50/HF
			Sufficient vitamin D	50 (56%)	38.2 ± 6.6	/LF/RMSDD
Nalbant et al. (2017)	Turkey	105	Deficient vitamin D	54 (27.5%)	27.8 ± 5.54	HR/QTc/SDNN/SDANN//PNN50
			Sufficient vitamin D	51 (13%)	28.8 ± 6.48	/RMSDD
Mann et al. (2013)	Canada	34	Deficient vitamin D	7 (29%)	37 ± 5	
			Sufficient vitamin D	27 (37%)	38 ± 3	HR/HF/LF

### 3.2 Quality Evaluation Results

The bias analysis and evaluation results of the 6 included studies are shown in Table 2. All studies scored ≥ 6 stars, which are high‐quality studies.

**TABLE 2:** Quality assessment of included studies.

Author	Newcastle‐Ottawa scale									Total
	Selection			Comparability			Outcome			
	a	b	c	d	e	f	g	h	i	
Denizhan	*	*	*	*	*	*	*	*		8
Bekdas	*	*	*	*	*	*	*	*		8
Mustafa	*			*	*	*	*	*	*	7
U˘gur Canpolat	*			*	*	*	*	*		6
Nalbant	*			*	*	*	*	*	*	7
Michelle C	*	*	*	*	*	*	*	*		8

### 3.3 Meta Analysis Results

#### 3.3.1 VitD Sufficient vs. VitD Deficiency

**FIGURE 2:**
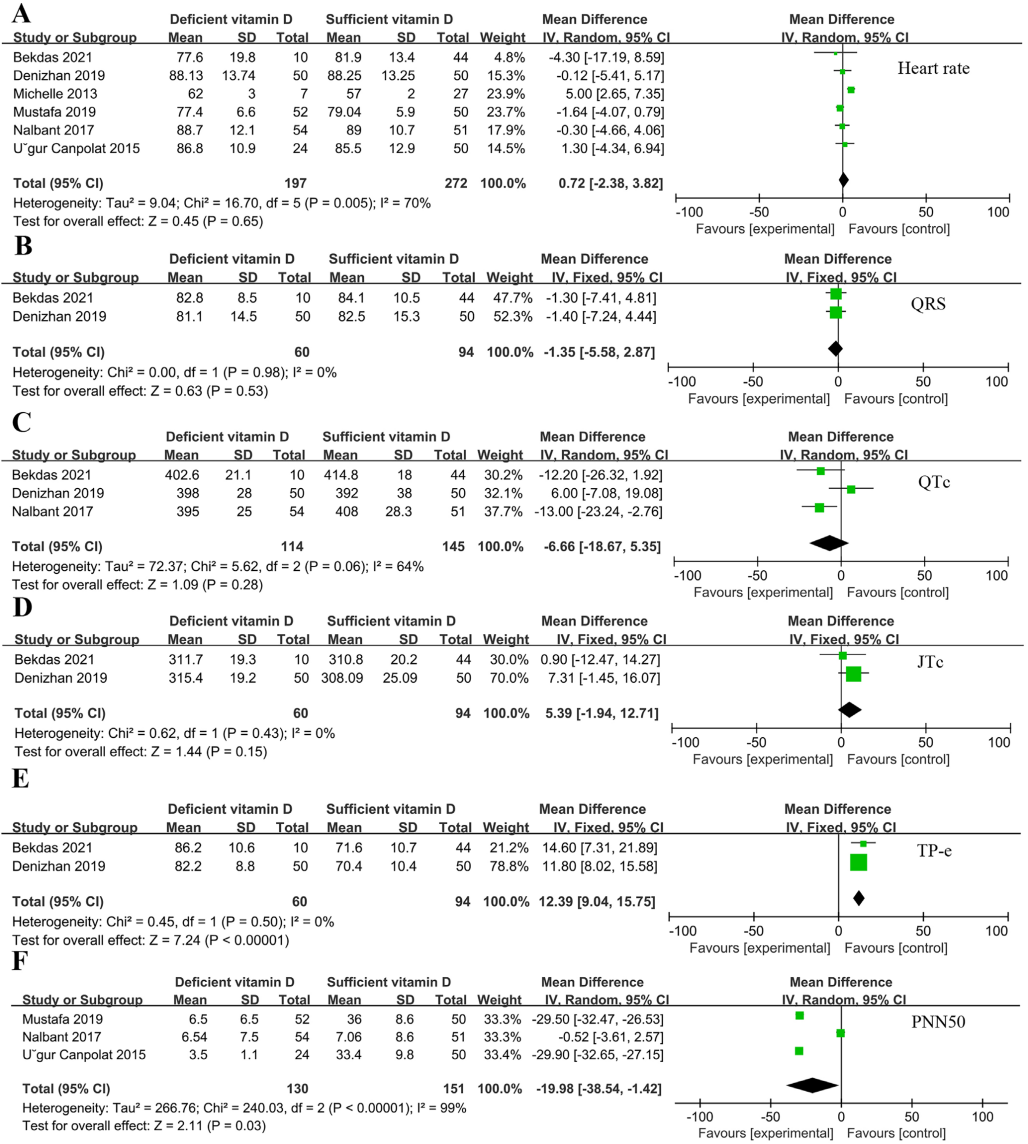
Forest plot of vitamin D sufficiency vs. vitamin D deficiency. (A) Heart rate. (B) QRS. (C) QTc. (D) JTc. (E) TP‐e. (F) PNN50.

**FIGURE 3:**
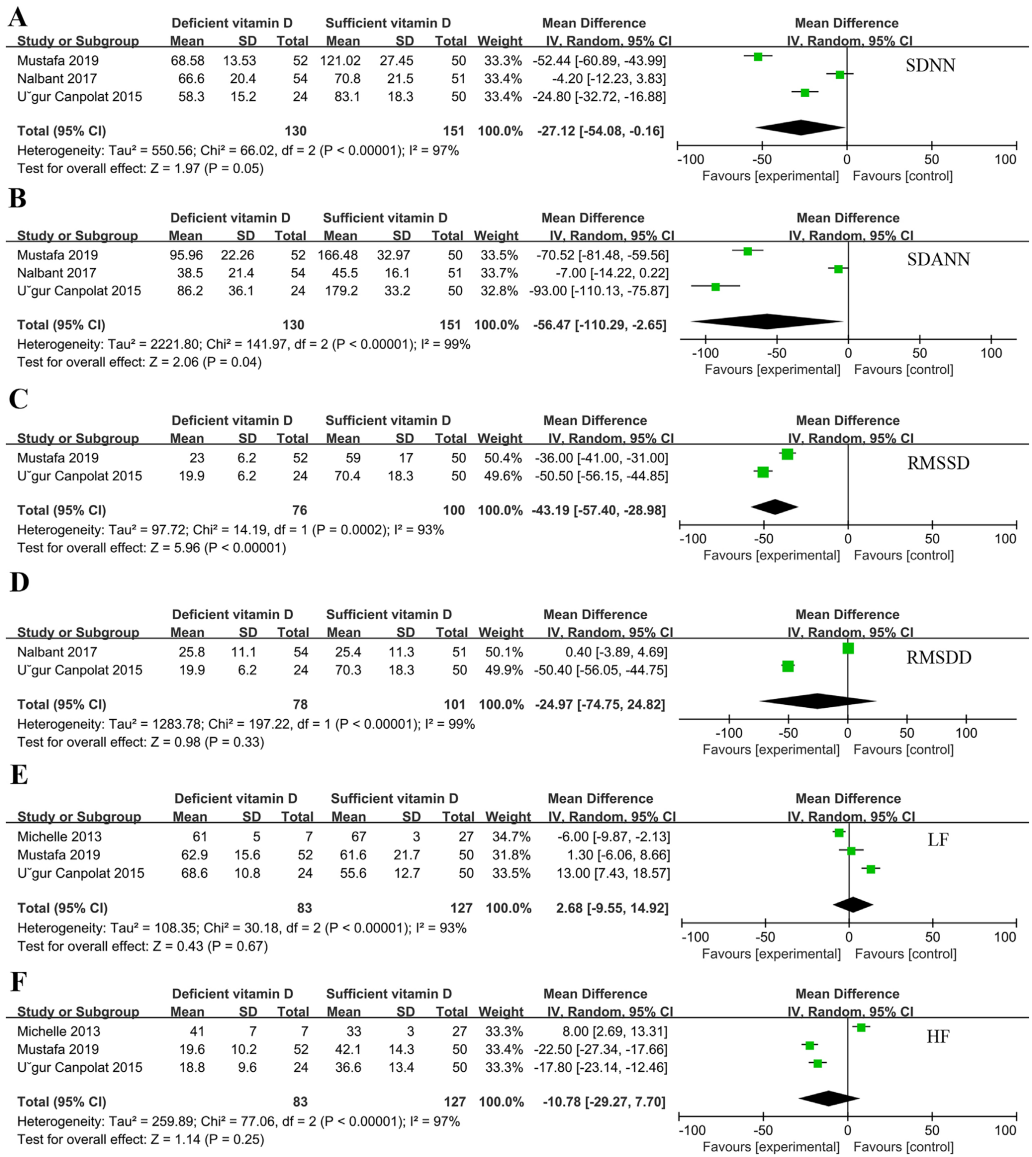
Forest plot of vitamin D sufficiency vs. vitamin D deficiency: (A) SDNN, (B) SDANN, (C) RMSSD, (D) RMSDD, (E) LF, (F) HF.

All 6 studies included both VitD‐deficient and VitD‐sufficient populations. A total of 496 individuals included 197 VitD deficient individuals and 272 VitD sufficient individuals. There was no significant difference in heart rate comparison between the two groups (*p* = 0.65) (Figure 2A). Two of the studies analyzed QRS (Figure 2B), JTc (Figure 2D), and RMSDD (Figure 3D) and showed that they were not associated with VitD levels (*p* > 0.05). Three of the studies analyzed QTc (Figure 2C), LF (Figure 3E), and HF (Figure 3F) and showed no significant difference between the two groups (*p* > 0.05). TP‐e (Figure 2E), PNN50 (Figure 2F), SDANN (Figure 3B), and RMSSD (Figure 3C) were significantly different between VitD‐deficient and VitD‐sufficient populations (*p* < 0.05). Except for TP‐e, VitD deficiency resulted in reduction of PNN50%, SDANN and RMSSD. Figure 3A shows the difference in SDNN between the two groups, the results show that VitD deficiency also reduce SDNN [MD = −27.12, 95% CI (−54.08, −0.16), *p* = 0.05].

**FIGURE 4:**
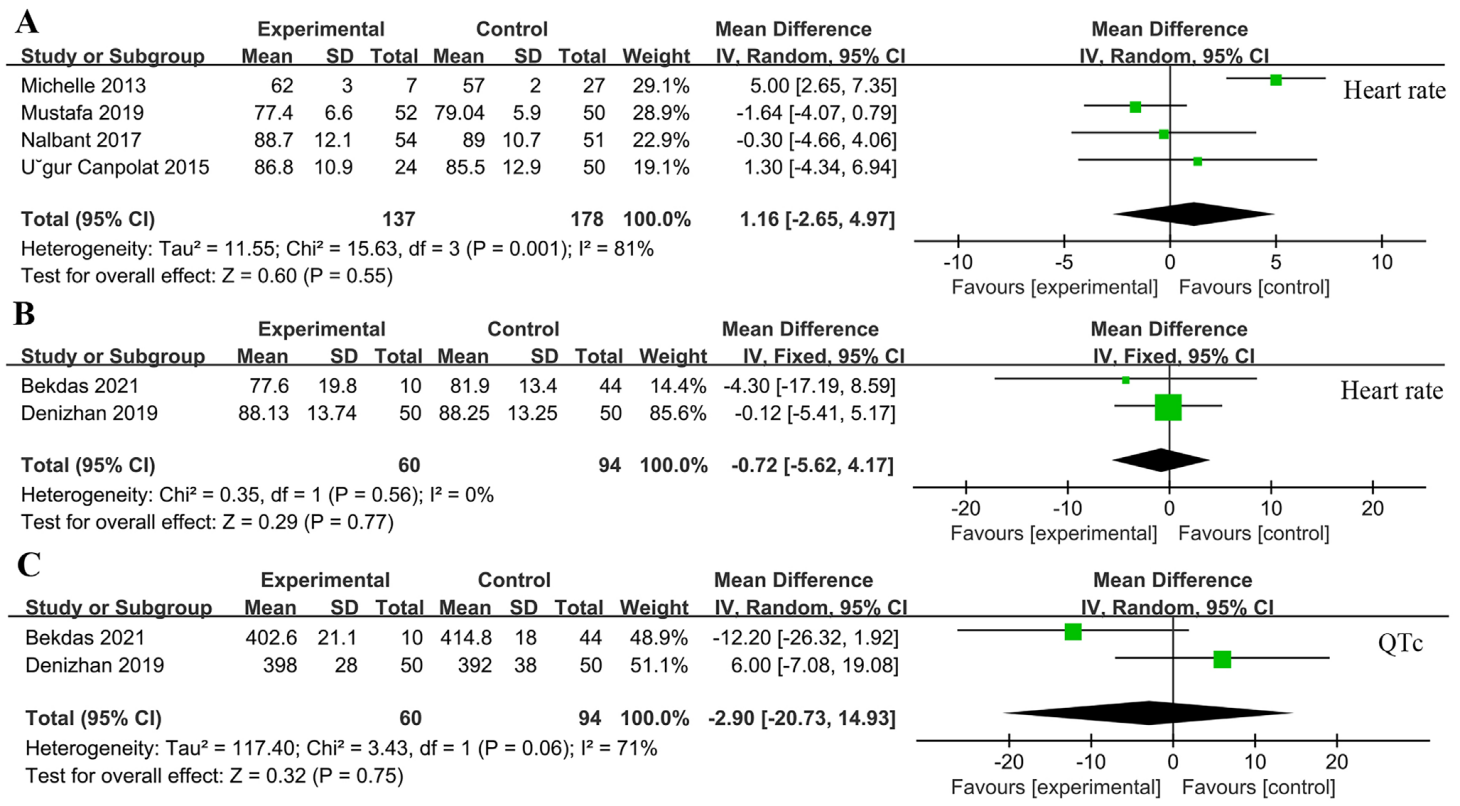
Results of heart rate and QTc subgroup analysis. (A) Heart rate in adults. (B) Heart rate in pediatrics. (C) QTc in pediatrics.

To exclude the influence of the apparent clinical heterogeneity of the population on the results, we performed a subgroup analysis of heart rate and QTc. The results showed that VitD deficiency compared to VitD sufficiency did not affect heart rate and QTc in either adults or pediatric populations (*p* > 0.05) (Figure 4).

#### 3.3.2 VitD Sufficient vs. VitD Insufficient

**FIGURE 5:**
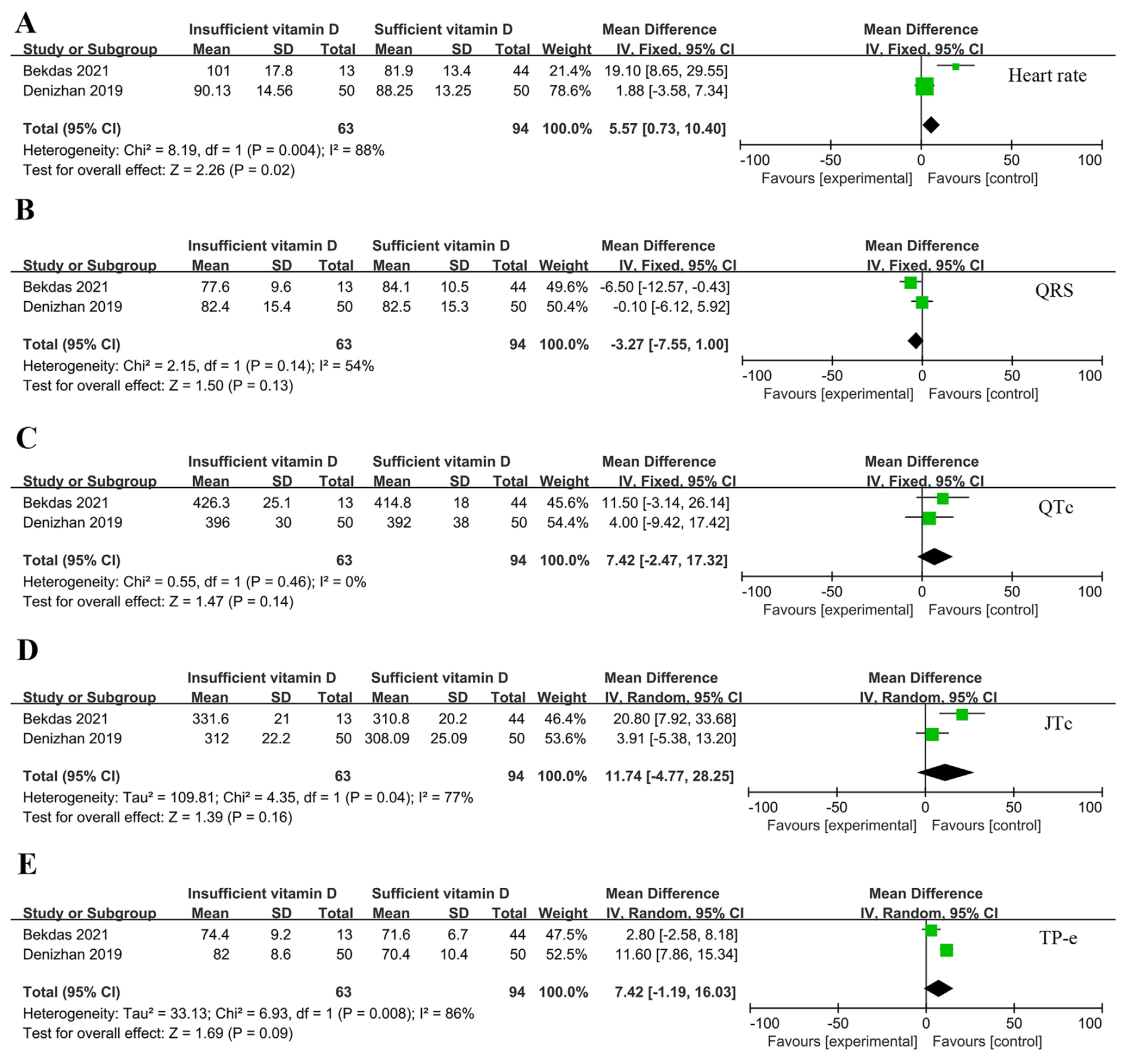
Forest plot of vitamin D sufficiency vs. vitamin D insufficiency. (A) Heart rate; (B) QRS; (C) QTc; (D) JTc; (E) TP‐e.

VitD sufficiency and insufficiency were examined in two investigations in terms of alterations in cardiac electrical activity. The study included 63 VitD insufficiency participants and 94 VitD sufficiency participants. There was no significant difference between the two groups in heart rate (Figure 5A), QRS (Figure 5B), QTc (Figure 5C), JTc (Figure 5D), and TP‐e (Figure 5E) (*p* > 0.05).

#### 3.3.3 VitD Insufficient vs. VitD Deficiency

**FIGURE 6:**
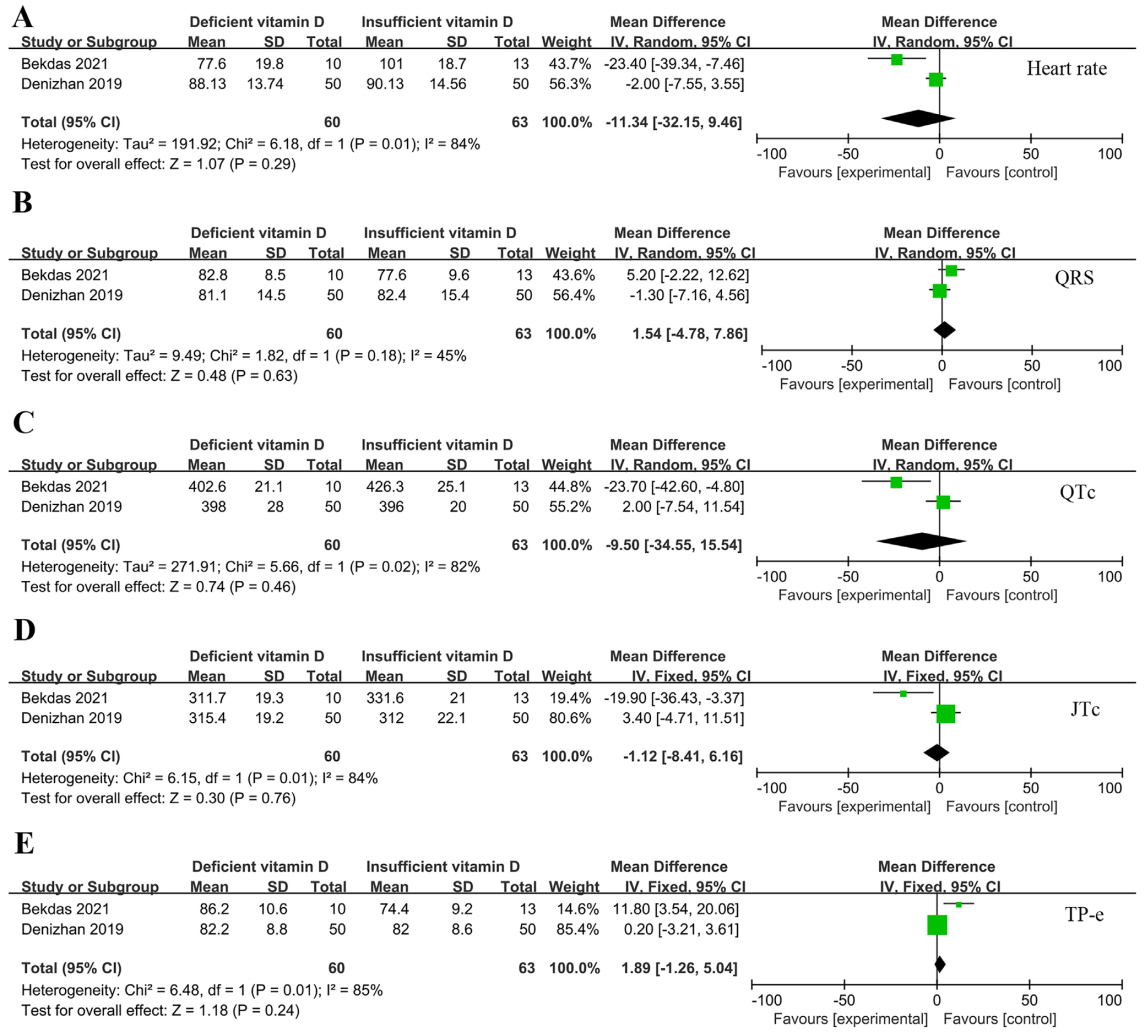
Forest plot of vitamin D deficiency vs. vitamin D insufficiency. (A) Heart rate. (B) QRS. (C) QTc. (D) JTc. (E) TP‐e.

Both investigations looked at a total of 123 people, 60 of whom were VitD deficient and 63 of whom were VitD insufficient. In terms of heart (Figure 6A), QRS (Figure 6B), QTc (Figure 6C), JTc (Figure 6D), and TP‐e (Figure 6E), there were no significant changes between VitD deficiency and VitD insufficiency persons (p > 0.05).

## 4 Discussion

This meta‐analysis found that in VitD deficiency people, PNN50, SDANN, and RMSSD were lower, and TP‐e was higher, compared to VitD‐sufficient people in the healthy population, with no significant changes in other electrocardiographic activity indices. Studies have shown that abnormal cardiac electrical activity increases the incidence of malignant arrhythmias and sudden cardiac death (Antzelevitch and Oliva 2006). As a result, the higher risk of aberrant cardiac electrical activity in VitD‐deficient healthy people compared to VitD‐sufficient healthy populations should lead to novel ideas for the prevention and early intervention of malignant arrhythmias and sudden cardiac death in these groups.

HRV has been demonstrated to be a useful predictor of sudden cardiac death and nauseating arrhythmic episodes in studies (Sun et al. 2021). A drop in HRV causes an increase in sympathetic tone, which might reduce the ventricular fibrillation threshold and is a risk factor; on the other hand, it causes an increase in parasympathetic tone, which is protective. A decrease in RMSDD may reflect cardiac autonomic dysfunction from an electrocardiographic perspective, with excessive sympathetic activation, thereby increasing the risk of arrhythmia. HRV is lowered in all individuals with coronary artery disease, and the degree of reduction is linked to the amount and severity of coronary atherosclerosis, and it is an independent predictor of coronary atherosclerosis (Skroza et al. 2020). HRV reduction is associated with left ventricular hypertrophy, cardiac insufficiency, and the occurrence of cardiac events in hypertensive individuals; the more severe the myocardial hypertrophy, the greater the HRV reduction and the higher and more severe the risk of ventricular arrhythmias (Díaz et al. 2021). Most people believe that time domain measurements like SDNN, SDANN, and PNN50 of less than 50 ms indicate much lower HRV and significantly higher morbidity and mortality. The present study showed a significant decrease in SDANN and PNN50 and a mild decrease in SDNN with VitD deficiency, suggesting that VitD deficiency may increase the morbidity and mortality of arrhythmias. Heart failure patients have damage to their autonomic nervous system, which results in a decrease in HRV, with both LF and HF much lower than normal, with HF falling more severely; hence, HRV variations may represent the severity of compromised cardiac function (Tsai et al. 2020). The present study shows that VitD deficiency does not affect LF and HF, implying unimpaired cardiac function. Changes in HRV, increased sympathetic activity, and decreased vagal activity may occur as a result of smoking, excessive alcohol use, and obesity, all of which have been linked to the development of cardiovascular disease (Strüven et al. 2021). In addition, research has demonstrated that patients with gestational diabetes mellitus (GDM) exhibit significantly prolonged atrial conduction times and increased P‐wave dispersion (PWD), suggesting potential underlying electrophysiological abnormalities (Kok et al. 2023).

The distance between the beginning of the QRS wave and the end of the T wave of the ECG can be utilized to anticipate malignant arrhythmias due to abnormalities in ventricular repolarization. QT interval, corrected QT interval (QTc), and QT dispersion (QTd) are some of the tests that can be used to detect ventricular repolarization problems. Tp‐e interval, which is the interval between the peak and endpoint of the T wave, has been employed as a novel indicator to diagnose ventricular repolarization abnormalities in recent years (Zhao et al. 2021). It better reflects changes in ventricular electrical activity than traditional indexes. We discovered no significant change in QTc between VitD deficient and VitD sufficient people in this study; however, TP‐e was longer. This shows that TP‐e is a better predictor of ventricular wall repolarization fragmentation than QTc, and that it is more closely linked to the onset of ventricular arrhythmias. In patients with coronary artery disease, cardiomyopathy, and cardiac insufficiency, studies have demonstrated that TP‐e has a good predictive value for the incidence of malignant arrhythmias (Akcay et al. 2021). It has been suggested that myocardial voltage‐related parameters, including the Tp‐e interval and the Tp‐e/QTc ratio, could serve as valuable indicators for assessing and stratifying the risk of high‐risk ventricular arrhythmias in diabetic patients with retinopathy (Coşgun et al. 2021). Thus, in healthy subjects, there is reason to believe that TP‐e can be used as a predictor of ventricular arrhythmias.

VitD and VitD receptors (VDR) are involved in a variety of organ activities. Large levels of VDRs have been found in the myocardium, suggesting that VitD can impact myocardial contraction and the dynamic equilibrium of calcium ions in the heart. Cardiovascular illnesses such as coronary heart disease, hypertension, and peripheral vascular disease can all be caused by a lack of vitamin D (Gholami et al. 2019). VitD insufficiency has also been linked to ion channel disorders and autonomic dysfunction, which could be linked to the fact that VitD deficiency can cause malignant arrhythmias and sudden cardiac death (Acharya et al. 2022; Canpolat, Yayla et al. 2015). In this study, VitD‐deficient healthy participants had lower HRV and a longer ventricular repolarization time than VitD‐sufficient healthy subjects, which could be linked to an increased risk of arrhythmias in VitD‐deficient healthy people.

There are several drawbacks to this study. Because there are fewer studies on the relationship between VitD and cardiac electrical activity in healthy people, more research is needed to confirm this finding. Although there are more indications of cardiac autonomic and ventricular repolarization, there is little research that compares them. Furthermore, lower HRV and extended ventricular repolarization only indicate the likelihood of ventricular arrhythmias and sudden cardiac death, but long‐term follow‐up studies are needed to confirm this finding.

## 5 Conclusion

In healthy subjects, VitD deficiency affects electrocardiographic activity by lowering PNN50, SDNN, SDANN, and RMSSD, modestly lowering SDNN, and lengthening TP‐e. VitD insufficiency does not appear to be associated with indicators of ventricular repolarization.

## Author Contributions

**Jinsen Wang:** formal analysis (equal), methodology (equal). **Xiaoyu Pan:** data curation (equal), formal analysis (equal). **Xueqing Zhang:** conceptualization (equal), data curation (equal). **Lin Yue:** investigation (equal), methodology (equal). **Jiangli Ban:** formal analysis (equal). **Lin Ren:** formal analysis (equal). **Xiaolan Jiang:** formal analysis (equal). **Shuchun Chen:** project administration (equal), supervision (equal), writing – review and editing (equal).

## Ethics Statement

The authors have nothing to report.

## Consent

The authors confirm that the work described has not been published before (except in the form of an abstract or as part of a published lecture, review, or thesis); that it is not under consideration for publication elsewhere; that its publication has been approved by all co‐authors, if any; that its publication has been approved (tacitly or explicitly) by the responsible authorities at the institution where the work is carried out. The authors agree to publication in the Journal indicated below and also to publication of the article in English by Springer in Springer's corresponding English‐language journal. The copyright to the English‐language article is transferred to Springer effective if and when the article is accepted for publication. The authors warrant that his/her contribution is original and that he/she has full power to make this grant. The authors sign for and accepts responsibility for releasing this material on behalf of any and all co‐authors. The copyright transfer covers the exclusive right to reproduce and distribute the article, including reprints, translations, photographic reproductions, microform, electronic form (offline, online) or any other reproductions of similar nature. After submission of the agreement signed by the corresponding authors, changes of authorship or in the order of the authors listed will not be accepted by Springer.

## Conflicts of Interest

The authors declare no conflicts of interest.

## Data Availability

The authors have nothing to report.

## References

[fsn370340-bib-0001] Acharya, P. , M. S. Safarova , T. Dalia , et al. 2022. “Effects of Vitamin D Supplementation and 25‐Hydroxyvitamin D Levels on the Risk of Atrial Fibrillation.” American Journal of Cardiology 173: 56–63. 10.1016/j.amjcard.2022.02.040. 35369930

[fsn370340-bib-0002] Akcay, M. , M. Coksevim , and M. Yenercag . 2021. “Effect of Ranolazine on Tp‐e Interval, Tp‐e/QTc, and P‐Wave Dispersion in Patients With Stable Coronary Artery Disease.” Journal of Arrhythmia 37, no. 4: 1015–1022. 10.1002/joa3.12549. 34386127 PMC8339098

[fsn370340-bib-0003] Antzelevitch, C. , and A. Oliva . 2006. “Amplification of Spatial Dispersion of Repolarization Underlies Sudden Cardiac Death Associated With Catecholaminergic Polymorphic VT, Long QT, Short QT and Brugada Syndromes.” Journal of Internal Medicine 259, no. 1: 48–58. 10.1111/j.1365-2796.2005.01587.x. 16336513 PMC1474026

[fsn370340-bib-0021] Bagrul, D. , and F. Atik . 2019. “Association of Vitamin D Deficiency With Ventricular Repolarization Abnormalities.” Kardiologia Polska 77, no. 9: 853–858. 10.33963/kp.14888. 31289258

[fsn370340-bib-0022] Bekdas, M. , M. Inanir , Z. Ilhan , and E. Ildes . 2021. “Effects of Serum Vitamin D Level on Ventricular Repolarization in Children and Adolescents.” Bratislavské Lekárske Listy 122, no. 11: 816–820. 10.4149/bll_2021_130. 34672674

[fsn370340-bib-0004] Canpolat, U. , Ç. Yayla , M. K. Akboğa , et al. 2015. “Effect of Vitamin D Replacement on Atrial Electromechanical Delay in Subjects With Vitamin D Deficiency.” Journal of Cardiovascular Electrophysiology 26, no. 6: 649–655. 10.1111/jce.12656. 25772677

[fsn370340-bib-0023] Canpolat, U. , F. Özcan , Ö. Özeke , et al. 2015. “Impaired Cardiac Autonomic Functions in Apparently Healthy Subjects With Vitamin D Deficiency.” Annals of Noninvasive Electrocardiology 20, no. 4: 378–385. 10.1111/anec.12233. 25363566 PMC6931811

[fsn370340-bib-0005] Coşgun, M. , İ. Sincer , T. Erdoğdu , et al. 2021. “Myocardial Repolarization Is Affected in Patients With Diabetic Retinopathy.” Journal of Surgery and Medicine 5, no. 7: 683–686. 10.28982/josam.955574.

[fsn370340-bib-0006] Díaz, H. S. , D. C. Andrade , C. Toledo , et al. 2021. “Inhibition of Brainstem Endoplasmic Reticulum Stress Rescues Cardiorespiratory Dysfunction in High Output Heart Failure.” Hypertension 77, no. 2: 718–728. 10.1161/hypertensionaha.120.16056. 33307852

[fsn370340-bib-0024] Dogdus, M. , S. Burhan , Z. Bozgun , et al. 2019. “Cardiac Autonomic Dysfunctions are Recovered With Vitamin D Replacement in Apparently Healthy Individuals With Vitamin D Deficiency.” Annals of Noninvasive Electrocardiology 24, no. 6: e12677. 10.1111/anec.12677. 31339201 PMC6931406

[fsn370340-bib-0007] Gholami, F. , G. Moradi , B. Zareei , et al. 2019. “The Association Between Circulating 25‐Hydroxyvitamin D and Cardiovascular Diseases: A Meta‐Analysis of Prospective Cohort Studies.” BMC Cardiovascular Disorders 19, no. 1: 248. 10.1186/s12872-019-1236-7. 31699030 PMC6836514

[fsn370340-bib-0008] Han, H. , S. I. Chung , H. J. Park , et al. 2021. “Obesity‐Induced Vitamin D Deficiency Contributes to Lung Fibrosis and Airway Hyperresponsiveness.” American Journal of Respiratory Cell and Molecular Biology 64, no. 3: 357–367. 10.1165/rcmb.2020-0086OC. 33296297

[fsn370340-bib-0009] Kok, Z. , I. Sincer , Y. Günes , and U. M. Ural . 2023. “Assessment of Atrial Conduction Time and P‐Wave Dispersion in Patients With Gestational Diabetes Mellitus.” International Journal of Diabetes in Developing Countries 43, no. 4: 538–543. 10.1007/s13410-022-01136-6.

[fsn370340-bib-0010] Lips, P. , K. D. Cashman , C. Lamberg‐Allardt , et al. 2019. “Current Vitamin D Status in European and Middle East Countries and Strategies to Prevent Vitamin D Deficiency: A Position Statement of the European Calcified Tissue Society.” European Journal of Endocrinology 180, no. 4: 23–54. 10.1530/eje-18-0736. 30721133

[fsn370340-bib-0011] Luo, X. , J. Xiong , H. Cai , et al. 2022. “Effects of Vitamin D Deficiency on the Function of the Cardiac Autonomic Nervous System in Rats.” Cardiovascular Therapeutics 2022: 4366948. 10.1155/2022/4366948. 35387268 PMC8967557

[fsn370340-bib-0025] Mann, M. C. , D. V. Exner , B. R. Hemmelgarn , et al. 2013. “Vitamin D Levels are Associated With Cardiac Autonomic Activity in Healthy Humans.” Nutrients 5, no. 6: 2114–2127. 10.3390/nu5062114. 23752493 PMC3725496

[fsn370340-bib-1006] Nalbant, A. , M. B. Vatan , P. Varım , C. Varım , T. Kaya , and A. Tamer . 2017. “Does Vitamin D Deficiency Effect Heart Rate Variability in Low Cardiovascular Risk Population?” Open Access Macedonian Journal of Medical Sciences 5, no. 2: 197–200. 10.3889/oamjms.2017.041. 28507628 PMC5420774

[fsn370340-bib-0012] Pines, A. 2014. “Vitamin D and Health Issues—Questioned Benefits.” Climacteric 17, no. 6: 657–659. 10.3109/13697137.2014.949232. 25203615

[fsn370340-bib-0013] Skroza, N. , A. Mambrin , I. Proietti , et al. 2020. “Evaluation of Cardiovascular Risk in Hidradenitis Suppurativa Patients Using Heart Rate Variability (HRV) Analysis.” Cardiovascular Therapeutics 2020: 1321782. 10.1155/2020/1321782. 32695226 PMC7349464

[fsn370340-bib-0014] Strüven, A. , C. Holzapfel , C. Stremmel , and S. Brunner . 2021. “Obesity, Nutrition and Heart Rate Variability.” International Journal of Molecular Sciences 22, no. 8: 4215. 10.3390/ijms22084215. 33921697 PMC8072942

[fsn370340-bib-0015] Sun, X. , S. Zhao , K. Chen , et al. 2021. “Association Between Cardiac Autonomic Function and Physical Activity in Patients at High Risk of Sudden Cardiac Death: A Cohort Study.” International Journal of Behavioral Nutrition and Physical Activity 18, no. 1: 128. 10.1186/s12966-021-01200-0. 34544427 PMC8454096

[fsn370340-bib-0016] Tiwari, R. , R. Kumar , S. Malik , T. Raj , and P. Kumar . 2021. “Analysis of Heart Rate Variability and Implication of Different Factors on Heart Rate Variability.” Current Cardiology Reviews 17, no. 5: e160721189770. 10.2174/1573403x16999201231203854. 33390146 PMC8950456

[fsn370340-bib-0017] Tsai, C. H. , H. P. Ma , Y. T. Lin , et al. 2020. “Usefulness of Heart Rhythm Complexity in Heart Failure Detection and Diagnosis.” Scientific Reports 10, no. 1: 14916. 10.1038/s41598-020-71909-8. 32913306 PMC7483411

[fsn370340-bib-0018] Yaman, B. , L. Cerit , H. K. Günsel , et al. 2020. “Is There any Link Between Vitamin D and Recurrence of Atrial Fibrillation After Cardioversion?” Brazilian Journal of Cardiovascular Surgery 35, no. 2: 191–197. 10.21470/1678-9741-2019-0166. 32369300 PMC7199985

[fsn370340-bib-0019] Yilmaz Coşkun, F. , G. Elboğa , G. Altunbaş , E. Vuruşkan , B. K. Uğur , and M. Sucu . 2018. “Evaluation of Ventricular Repolarization Features With Tp‐e, Tp‐e/QTc, JTc and JTd During Electroconvulsive Therapy.” Journal of Electrocardiology 51, no. 3: 440–442. 10.1016/j.jelectrocard.2018.02.003. 29477501

[fsn370340-bib-0020] Zhao, D. , B. Liang , J. Peng , et al. 2021. “Tp‐e and (Tp‐e)/QT Ratio as a Non‐Invasive Risk Factors for Malignant Ventricular Arrhythmia in Patients With Idiopathic Ventricular Premature Complexes.” Journal of Clinical Laboratory Analysis 35, no. 2: e23636. 10.1002/jcla.23636. 33332643 PMC7891518

